# Surgical delays between indication and operating room access in patients undergoing glaucoma filtration surgery

**DOI:** 10.1038/s41598-026-39121-2

**Published:** 2026-02-09

**Authors:** Luca Agnifili, Matteo Sacchi, Michele Figus, Michele Iester, Antonio Pinna, Chiara Posarelli, Paola Cassottana, Luca Virgilio Corboli, Matteo Fornaro, Alessandro Palma, Annalisa Marotta, Marta di Nicola, Leonardo Mastropasqua

**Affiliations:** 1 Ophthalmology Clinic, Department of Medicine and Ageing Science, University ‘G. d’Annunzio’ of Chieti-Pescara, Via dei Vestini, Chieti (CH), 66100 Italy; 2 Ophthalmology, Azienda Ospedaliero-Universitaria di Sassari, Sassari, Italy; 3 Ophthalmology Unit, Department of Surgery, Medicine, Molecular and Emergency, University of Pisa, Pisa, Italy; 4 Department of Neuroscience, Rehabilitation, Ophthalmology, Genetics, Maternal and Child Health (DiNOGMI), Eye Clinic, University of Genova, Genova, Italy; 5 Department of Medical, Oral and Biotechnological Sciences, Laboratory of Biostatistics, University ‘G. d’Annunzio’ of Chieti-Pescara, Chieti, Italy

**Keywords:** Glaucoma filtration surgery, Waiting times, Surgical delays, Pre-surgical workup, Pre-surgical pathway, Glaucoma, Risk factors, Surgery

## Abstract

Surgical delays between indication for surgery and access to the operating room in glaucoma filtration surgery (GFS) are unknown. We reviewed medical charts of the first fifty patients’ undergoing GFS from February 2017, 2019, and 2022 with the aim to: (i) measure waiting times between indication for surgery to pre-surgical workup, pre-surgical workup to surgery, and indication to surgery; (ii) identify factors affecting the pre-surgical path duration, and (iii) evaluate whether waiting times changed in the 2017–2022 quinquennium.

633 patients, in four tertiary-care Italian Centers, were enrolled. At the indication for surgery, the mean deviation (MD) was − 13.4 dB (IQR: -21.2; -6.9), with an advanced glaucoma in 54.6% of cases (MD: -20.3 dB), and a median IOP of 24 mmHg (IQR: 20.0–28.0). Overall, patients waited 44.0 days (IQR: 21.0–72.0) between indication for GFS and surgery, with the interval between indication and pre-surgical workup being the most time consuming step (32.0 days (IQR: 8.0-51.8)). Patients living in South Italy, with primary glaucoma, an IOP less than 20 mmHg, scheduled for a first phaco-combined trabeculectomy, non-monocular, and with systemic comorbidities, waited more. Since glaucoma may continue to progress while waiting, efficient organizational strategies should be adopted to optimize the pre-surgical path duration.

## Introduction

Lowering the intraocular pressure (IOP) represents the only proven therapy to preserve visual function in glaucoma^1^. In the majority of cases, the initial approaches to lowering IOP in a stepwise therapeutic strategy are medication and laser^1,2^. When target IOP is not achieved with medications and damage progression continues at an unsafe rate, surgery is required^2,3^. Once a patient receives indication for surgery, he undergoes a multi-step preparation path that brings him into the operating room after a certain wait.

These steps include the insertion of the patient in a waiting list, the pre-surgical workup, the wait after preparation, and the final access to the operating room. The duration of these steps depends on: (i) the level of priority given to the condition, which in turn depends on the IOP value, stage of disease, rate of progression, and presence of comorbidities; (ii) the type of surgery: minimally invasive bleb forming (MIBS) surgery, standard glaucoma filtration surgery (GFS) (trabeculectomy), bleb revision (MIBS or primary GFS), second GFS, or glaucoma drainage device implantation; (iii) the availability of consultation rooms, diagnostic platforms, ophthalmologists, and nurses; (iv) the availability of routine surgical supplies including filtration devices; and (v) the availability of trained glaucoma surgeons, anesthetists, and operating rooms. When the duration of the entire pre-surgical step is excessively long, the optic nerve damage may continue to progress. Therefore, the surgical delays times could represent an additional risk factor for further visual function loss^4–6^. To date, we are not aware of previous studies that investigated these aspects. Therefore, the aims of the present study were: (i) to measure the waiting times between pre-surgical steps in four glaucoma-specialized Italian academic centers; (ii) to identify factors that affect the duration of the pre-surgical path; and (iii) to evaluate whether the duration of pre-surgical steps has changed during the last five years. There are no previous studies that investigated the waiting time from indication to access into the operating room for patients candidate to glaucoma filtration surgery.

## Results

633 patients met the inclusion criteria. Gender, and age, geographical provenance of patients, and temporal distribution of surgeries are reported in Table 1.

At the time of surgical indication, 91% of patients were receiving maximal topical therapy (three or more topical medications) while awaiting surgery, and 69% were treated with oral acetazolamide. Acetazolamide was prescribed selectively according to IOP levels, comorbidities, and drug tolerability.

Overall, 524 (82.8%) cases were first surgeries with 239 trabeculectomies, 260 MIBS, and 25 tubes, whereas 109 (17.2%) were reinterventions (Table 2).

**Fig. 1:**
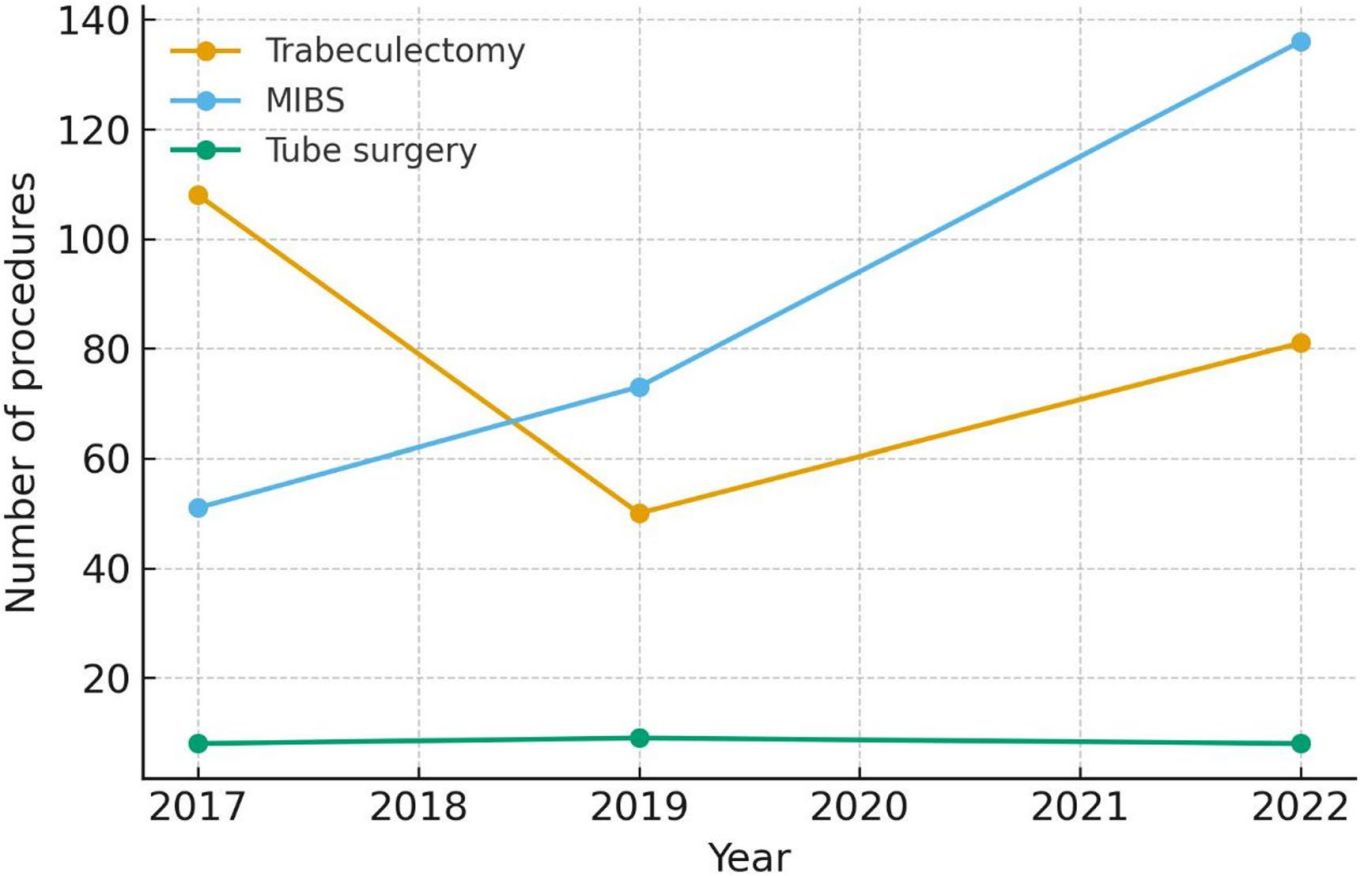
Temporal trends in glaucoma surgical procedures between 2017 and 2022. The figure illustrates temporal trends in the number of surgical procedures performed between 2017 and 2022. While the number of trabeculectomies decreased over time, minimally invasive bleb-forming surgery (MIBS) increased markedly, and the volume of tubes remained stable throughout the study period.

During the quinquennium analyzed, the number of tubes did not change, trabeculectomies decreased from 108 (2017) to 81 (2022), while the number of MIBS increased from 51 (2017) to 136 (2022) (Fig. 1). At the indication for surgery, the entire sample had an MD of -13.4 dB (IQR: -21.2; -6.9), a VFI of 66% (IQR: 32%-89%), and an IOP of 24 mmHg (IQR: 20.0–28.0). According to MD, 111 patients (22.0%) had an early stage of glaucoma (-2.60 dB (IQR: -3.7- -1.5)), 118 (23.4%) a moderate stage (-8.9 dB (IQR: -10.6- -7.7)), and 275 (54.6%) an advanced stage (-20.3 dB (IQR: -24.6- -16.5). According to VFI, 199 patients (60.5%) had values > 50%, whereas 130 patients (39.5%) < 50%. According to baseline IOP, 156 patients (26.8%) had an IOP < 20 mmHg (18 mmHg), 339 (58.0%) between 20 and 30 mmHg (25 mmHg), and 89 patients (15.2%) > 30 mmHg (36 mmHg).

### Waiting times between indication for surgery and date of surgery

**Fig. 2:**
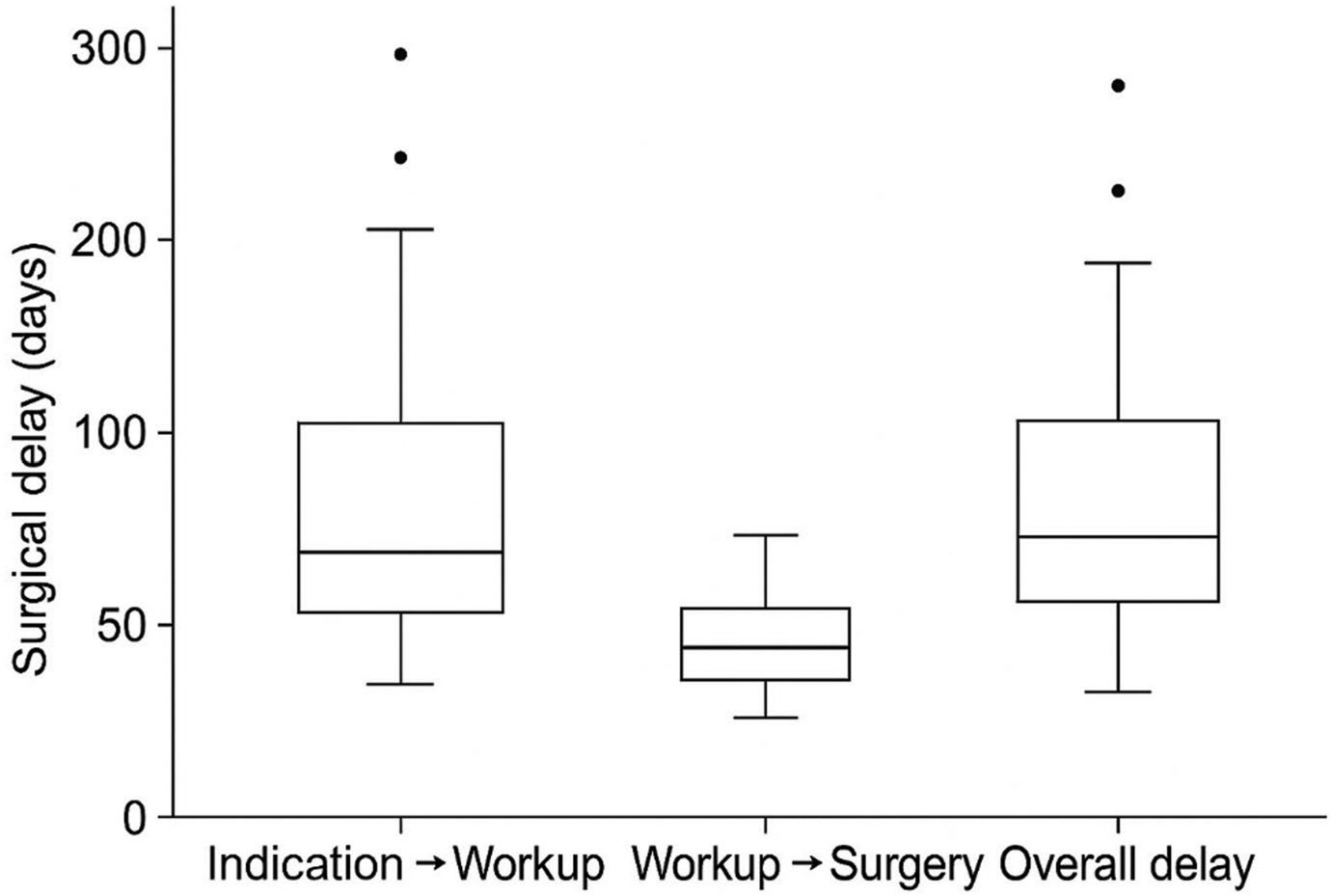
Box-whiskers plots representing the three pre-surgical time gaps (indication to pre-surgical workup (**A**), pre-surgical workup to surgery (**B**) and indication to surgery (**C**)). The boxes represent the 25th -75th percentiles range, the horizontal line the median. Error bars above and below the boxes indicate the 90th and 10th percentiles, respectively. The median time gap was 32.0 days (IQR: 8.0-51.8) between indication to pre-surgical workup, 7.0 days (IQR: 4.0–19.0) between pre-surgical workup for surgery and 44.0 days (IQR: 21.0–72.0) between indication for surgery.

Overall, patients waited 44.0 (IQR: 21.0–72.0) days between the first indication for surgery and the date of surgery. The median waiting time was 32.0 days (IQR: 8.0-51.8) between indication and the pre-surgical workup, and 7.0 days (IQR: 4.0–19.0) between pre-surgical workup and the date of surgery (Fig. 2).

### Factors that affected the duration of the pre-surgical path

**Fig. 3:**
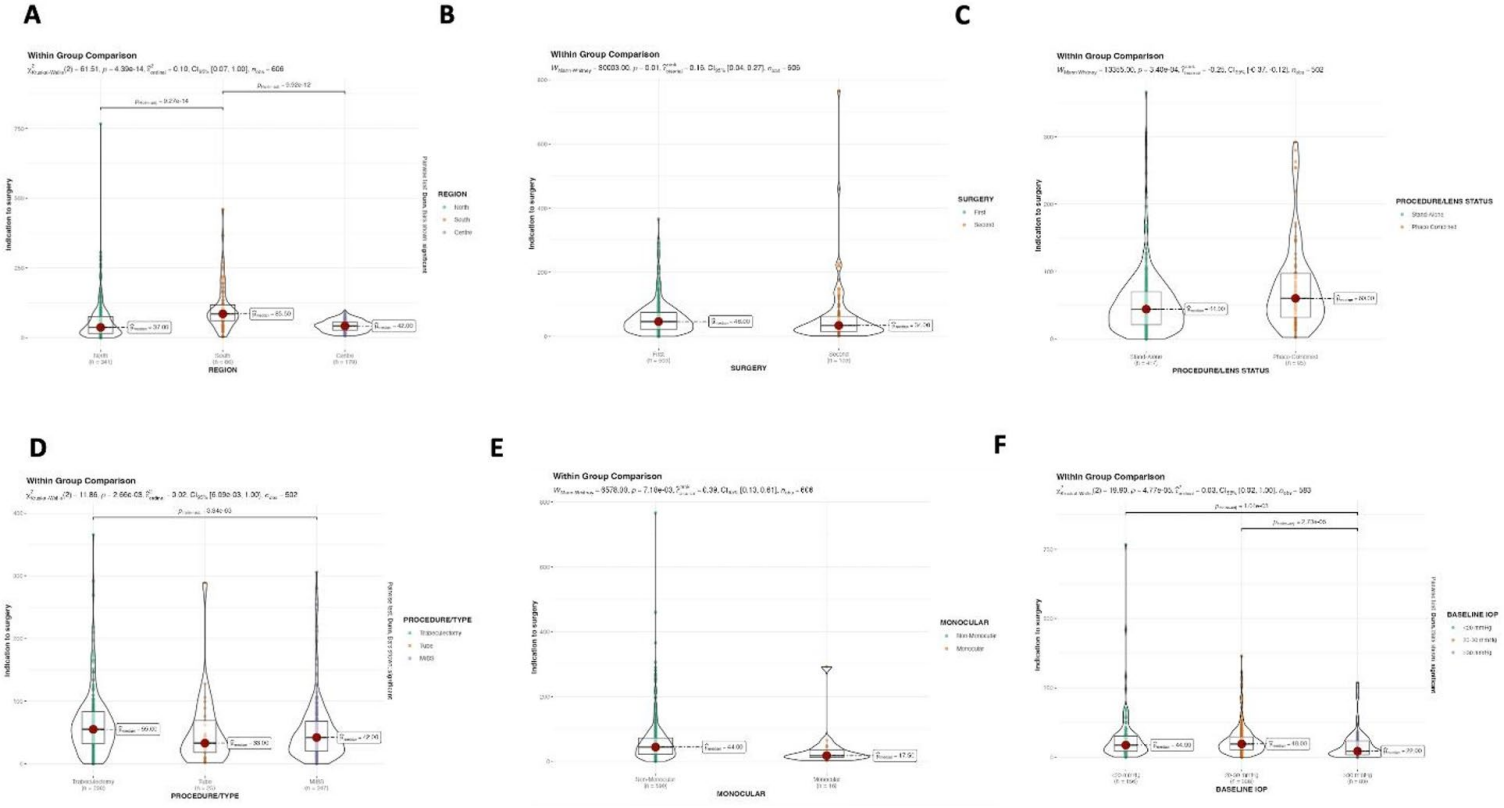
Violin plots. Within group comparison for factors affecting the waiting time “indication to surgery” (**A**–**F** geographical origin; first/second surgery; procedure/lens status; type of procedure; presence/absence of monocularity; baseline intraocular pressure). P-value reported in figures are relative to non-parametric pairwise comparisons.

For the *number of surgeries*, patients who underwent a second surgery had a faster pre-surgical path compared to those candidates to a first surgery (25% shorter) (Table 2; Fig. 3). For the *geographic distribution*, waiting times were about 50% longer in South Italy, especially when compared with northern Centers (Table 1; Fig. 3). For *glaucoma phenotype*, the temporal step between pre-surgical workup and surgery was shorter in secondary compared to primary glaucoma (Table 2; Fig. 3).

For *surgical technique*, patients undergoing MIBS waited the shortest times in both steps, whereas those scheduled for trabeculectomy waited the longest times (Table 2; Fig. 3). *Phaco-combined procedures* had a significantly longer wait (one third more) between the date of indication and the date of surgery compared to stand-alone approaches (Table 2; Fig. 3). As expected, waiting times in *monocular* patients were significantly shorter compared to non-monocular patients, with the entire pre-surgical path lasting about one month less (Table 2; Fig. 3). Patients with *systemic comorbidities* waited a longer time between pre-surgical workup and surgery (Table 2; Fig. 3). For *baseline IOP*, patients with an IOP > 30 mmHg had a wait two times shorter than patients with an IOP < 30 mmHg (22 vs. 44 days, respectively) (Table 2; Fig. 3).

Unexpectedly, the *perimetric stage* of the disease did not correlate with any of the waiting times (Table 2; Fig. 3). However, though unsignificant, a certain trend for shorter waits was noted with an MD<-12 dB or with a VFI < 50%. The BCVA did not worsen during the wait, with values of 0.567 logMar (SD 0.290) at the surgical indication and 0.569 logMar (SD 0.291) at the pre-surgical workup (Wilcox U test *p* < 0.001).

### Differences in waiting times in the last quinquennium

The univariate analysis did not show significant differences among the three time points for the three considered waiting times (Table 1).

The multivariate analysis confirmed the significant relationships between the waiting time “*indication to surgery*” and some factors that affected the duration of the pre-surgical path such as living in the South Italy, undergoing a first phaco-combined trabeculectomy, having a baseline IOP less than 20 mmHg or systemic comorbidities (Table 3).

A trend to statistical significance was noted for patients undergoing surgery in 2022, who waited a little more with respect to 2017.

**Table 1:** Demographic factors affecting the duration of the pre-surgical pathway.

Variable	*n* (%)	Time gap (days)		
		Indication to pre-surgical workup	Pre-surgical workup to surgery	Indication to surgery
Age < 70 years	289 (45.7)	35.0 (7.2–55.8)	8.0 (4.0–21.0)	45.0 (22.0–74.0)
Age ≥ 70 years	344 (54.3)	30.0 (8.0–49.0)	7.0 (4.0-16.3)	43.0 (20.0–70.0)
p-value		*p* = 0.489	*p* = 0.471	*p* = 0.304
Gender: Male	329 (56.4)	35.0 (14.0–53.0)	8.0 (4.5–21.0)	49.0 (27.0–77.0)
Female	254 (43.6)	35.0 (11.0–56.0)	7.0 (4.0–15.0)	44.0 (22.0–74.0)
p-value		*p* = 0.612*	*p* = 0.244*	*p* = 0.290*
Year: 2017	161 (25.4)	29.00 (9.5–48.5)	7.0 (5.0–14.0)	42.0 (20.5–63.0)
2019	200 (31.6)	34.5 (5.0-59.8)	7.0 (3.0–22.0)	47.0 (20.0–77.0)
2022	272 (43.0)	33.0 (12.0-49.3)	8.0 (4.0–21.0)	44.0 (23.0–79.0)
p-value		*p* = 0.491^§^	*p* = 0.395^§^	*p* = 0.081^§^
Region: North Italy	179 (28.3)	18.0 (4.0–53.0)	9.0 (3.0–19.0)	37.0 (15.0–76.0)
Centre	342 (54.0)	37.0 (21.25–49.75)	5.0 (4.0–7.0)	42.0 (27.0–56.0)
South	112 (17.7)	36.5 (26.0-50.5)	42.0 (27.0–64.0)	85.5 (61.0-117.8)
p-value		*p* < 0.001^**§**^	*p* < 0.001^**§**^	*p* < 0.001^**§**^

Data are expressed as median and interquartile range (Q1-Q3).

*p-value derived from Mann-Whitney U test.

^§^ p-value derived from Kruskal-Wallis test.

**Table 2:** Surgical- and clinical-related factors affecting the duration of the pre-surgical pathway.

Variable	*n* (%)	Waiting times (days)		
		Indication to pre-hospitalization	Pre-hospitalization to surgery	Indication to surgery
First surgery	524 (82.8)	35.0 (11.0–53.0)	8.0 (4.0-18.5)	46.0 (22.0–74.0)
Second surgery	109 (17.2)	16.0 (5.0–46.0)	6.0 (3.75-20.0)	34.0 (15.5–62.5)
p-value		p = 0.007 *	*p* = 0.130 *	p = 0.011 *
Stand-Alone Procedure	542 (85.8)	30.5 (7.7–50.0)	7.0 (4.0–16.0)	42.0 (20.0–69.0)
Phaco-Combined Procedure	90 (14.2)	37.0 (12.0-66.3)	10.5 (6.0–28.0)	60.0 (31.5–95.5)
p-value		p = 0.042 *	p < 0.001 *	p < 0.001 *
Procedure at first surgery				
Tube	25 (4.8)	21.0 (6.0–56.0)	7.0 (3.0–14.0)	33.0 (18.0–69.0)
Trabeculectomy	239 (45.6)	37.0 (14.0-51.2)	8.0 (4.0–23.0)	55.0 (32.0–83.0)
MIBS	260 (2.0)	31.5 (6.7–48.2)	7.0 (4.7–15.0)	42.0 (20.5–68.0)
p-value		*p* < 0.072 ^§^	*p* < 0.173 ^§^	*p* < 0.003 ^§^
Procedure at second surgery				
Tube	14 (12.8)	62.0 (24.2–136.0)	7.0 (3.5–12.0)	80.5 (46.2-139.2)
Trabeculectomy	31 (28.4)	10.0 (3.5–51.2)	11.0 (5.2–27.7)	35.0 (16.2–70.0)
MIBS	35 (32.1)	15.0 (1.5–46.0)	5.0 (2.0-20.5)	34.0 (8.0-52.5)
p-value		*p* < 0.021 ^§^	*p* < 0.140 ^§^	*p* < 0.013 ^§^
Primary Glaucoma	515 (81.4)	32.0 (7.0–51.0)	8.0 (4.0–22.0)	45.0 (21.0–76.0)
Secondary Glaucoma	118 (18.6)	32.0 (11.75–56.25)	6.0 (4.0-9.2)	41.0 (22.0–67.0)
p-value		*p* = 0.457 *	p = 0.004 *	*p* = 0.474 *
Monocular	16 (2.5)	5.5 (1.0-26.8)	11.5 (7.5–13.5)	17.5 (12.0–35.0)
Non-Monocular	617 (97.5)	33.0 (8.0–53.0)	7.0 (4.0–20.0)	44.0 (22.0–72.0)
p-value		p = 0.005 *	*p* = 0.535 *	p = 0.007 *
Local Comorbidities	95 (15.0)	34.0 (5.5–68.0)	8.0 (2.5–15.0)	44.0 (20.0-84.5)
No Local Comorbidities	538 (85.0)	31.5 (8.0–50.0)	7.0 (4.0-20.3)	44.0 (21.5–70.0)
p-value		*p* = 0.670 *	*p* = 0.450 *	*p* = 0.406 *
Systemic Comorbidities	220 (34.8)	31.0 (6.0–60.0)	9.0 (5.0–21.0)	47.5 (20.2–81.0)
No Systemic Comorbidities	413 (65.2)	32.5 (10.0–49.0)	6.0 (4.0-15.8)	43.0 (21.7–69.3)
p-value		*p* = 0.967 *	p < 0.001 *	*p* = 0.173 *
IOP > 30 mmHg	89 (15.2)	15.5 (4.0-41.3)	6.0 (2.0–11.0)	22.0 (9.0–59.0)
20 ≤ IOP ≤ 30 mmHg	339 (58.0)	35.0 (13.0-52.8)	8.0 (5.0–22.0)	48.0 (27.0-73.8)
IOP < 20 mmHg	156 (26.7)	33.0 (8.0-63.3)	6.0 (4.0-16.5)	44.0 (21.7–76.3)
p-value		p = 0.003 *	p = 0.001 *	p < 0.001 *
VFI < 50%	130 (39.5)	37.0 (15.0–65.0)	10.0 (5.0-28.8)	60.0 (29.2–94.0)
VFI ≥ 50%	199 (60.5)	23.0 (43.0–61.0)	10.0 (5.0-27.3)	65.0 (39.0–88.0)
p-value		p = 0.168 *	p = 0.952 *	*p* = 0.429 *
MD ≤ − 12 dB	275 (54.6)	35.0 (13.0-51.5)	7.0 (4.0–19.0)	48.0 (23.0–76.0)
-12 ≤ MD ≤ -6 dB	118 (23.4)	38.0 (16.5–59.5)	7.0 (4.0-16.5)	50.5 (29.0–72.0)
MD > − 6 dB	111 (22.0)	40.0 (16.5–59.0)	8.0 (4.0–22.0)	56.0 (33.0–82.0)
p-value		p = 0.191 *	p = 0.884 *	p = 0.298 *

Data are expressed as median and interquartile range (Q1-Q3).

* p-value derived from Mann-Whitney U test.

^§^ p-value derived from Kruskal-Wallis test.

MIBS: Minimally Invasive Bleb-forming Surgery.

IOP: intraocular pressure (mmHg); VFI: visual field index (%); MD: mean deviation (dB).

**Table 3:** Multivariate linear regression analysis on indication to surgery waiting time.

Predictor	Coefficient (95%CI)	*p*-value
REGION: *North Italy*		
*South*	57.9 (41.8; 74.1)	***< 0.001***
*Centre*	5.2 (-7.2; 17.5)	*0.411*
PROCEDURE: *Stand-Alone*		
*Phaco-Combined*	22.8 (8.9; 36.7)	***0.001***
SYSTEMIC COMORBIDITIES: *No*		
*Yes*	14.6 (4.0; 25.1)	***0.007***
YEAR: *2022*		
*2019*	0.3 (-11.0;11.6)	*0.959*
*2017*	-12.2 (-24.2; -0.3)	***0.045***
SURGERY: *First*		
*Second*	-22.6 (-37.5; -7.7)	***0.003***
PROCEDURE *(first): Trabeculectomy*		
*Tube*	-11.6 (-8.7; 31.8)	*0.263*
*MIBS*	-10.7 (-21.0; -0.5)	***0.040***
IOP *> 30 mmHg*		
*20 ≤ IOP ≤ 30 mmHg*	11.7 (-1.6; 25.0)	*0.084*
*IOP < 20 mmHg*	23.9 (9.0; 38.8)	***0.002***

Model adjusted for age, monocular and primary/secondary glaucoma.

IOP: intraocular pressure.

MIBS: minimally invasive bleb-forming surgery.

## Discussion

The waiting time for surgery has been largely investigated across different specialties since may potentially lead to a huge burden for healthcare systems^7^. In ophthalmology, this issue has been examined for cataract surgery, but not for glaucoma^8–12^. In this disease, this aspect appears even more crucial since an excessive duration of the pre-surgical path may expose the patient to the risk of a further visual function loss.

In our study we found that patients candidate to GFS waited forty days to access the operating room, with the most time-consuming step being the pre-surgical workup, which was more than four times longer than the wait between pre-surgical workup and surgery. The most important reason determining the dilation of this step could be the excessively long waiting lists in our Centers which, being tertiary care structures, serve as surgical referral for vast geographical areas.

We found that there are some conditions that extended the pre-surgical path duration, such as living in the South (South areas have less resources in Italy), being affected with primary glaucoma, having a contained baseline IOP, needing a phaco-combined trabeculectomy, being non-monocular and with systemic comorbidities. The presence of these conditions led to waiting times that were three-four weeks longer.

In more detail, the longer waiting times for phaco-combined GFS could depend on the fact that more complex procedures require the availability of a more trained surgeon. A sort of “less felt urgency” can be hypothesized for the prolonged waiting times in patients with a primary form of glaucoma, low baseline IOP, and non-monocular. Conversely, a shorter waiting time was observed for reinterventions. A possible explanation is that these patients often present with a more complex or unstable clinical condition, prompting clinicians to prioritize surgery.

Patients with systemic comorbidities waited little more after pre-surgical workup probably because of the need of additional consultations.

Surprisingly, the age and the stage of disease did not affect the duration of the pre-surgical path. We may suppose that the IOP levels guide clinicians much more than the perimetric stage in establishing surgical priority. This indicates that, unfortunately, many ophthalmologists continue considering the IOP as the unique discriminant factor to proceed with surgery.

An additional aim of this study was to investigate whether during the last quinquennium, which hosted the major diffusion of new surgical procedures, there was a modification in the duration of the pre-surgical path. With a partial surprise, we did not observe any variation. In fact, although the entire waiting time for MIBS was shorter compared to standard GFS (41 vs. 53.5 days), the duration of the pre-surgical path did not decrease in the third period (2022), when the number of MIBS markedly increased (+ 37%) and standard GFS decreased (-25%) compared to 2017.

In our cohort, minimally invasive surgical procedures (minimally invasive glaucoma surgery (MIGS) and MIBS) constituted a substantial proportion of surgeries (260 cases, comparable to 239 trabeculectomies), and increased markedly over the quinquennium (from 51 to 136 procedures). This trend reflects the current evolution of glaucoma surgical practice.

Conversely, the multivariate analysis showed a trend for a longer wait in 2022. These results probably indicate that the availability of faster surgical procedures permit an increasing number of surgeries, thus satisfying the needs of a higher number of patients. But on the other hand, fast procedures cannot shorten the waiting times since favor an increase in demand. In fact, we registered a marked increase in the overall number of GFS in the quinquennium analyzed, with 161 filtration surgeries in 2017 and 272 in the 2022 (+ 40%).

Another remarkable finding was that most of patients (54.6%) underwent surgery in an advanced/severe stage of disease (MD: -20.26 dB). This indicates that in our Country GFS still remains the very last therapeutic resort, despite the advancements in terms of safety and duration of surgical procedures. Though a positive trend in the last quinquennium was observed, there were not significant differences in the baseline MD, with values of -14.46 dB, -13.47 dB, and − 13.10 dB, in the 2017, 2019 and 2022, respectively.

The present study presents several limitations. First, we are unable to assess the impact of prolonged surgical delays on the rate of damage progression since visual fields were only performed at the date of indication for surgery. Thus, this aspect limits the ability to evaluate the real clinical impact of delays. Further prospective studies including visual fields and OCTs (retinal nerve fiber layer and ganglion cell analysis) at pre-surgical workup and at the day of surgery are needed to address this question. Second, the number of surgeons, nurses, operating rooms, surgical sessions, and waiting lists were not considered. This is important, since strategies such as increasing funding to allocate more resources for operating rooms and additional staff, should be evaluated also in glaucoma^13–16^.

Third, our study involved only tertiary care, glaucoma-specialized, public, and academic healthcare centers. Thus, these results cannot be extended to other healthcare realities.

Fourth, we are not aware if at the moment of the indication for surgery, clinicians indicated a class of priority in accordance with the patient status. This is an important topic since a refinement of prioritization systems is considered of great relevance to address the issue of the delayed care^16^. In fact, in general surgery, prioritization protocols based on the severity of disease and patient-reported outcomes, have been reported to successfully shorten the surgical delay for patients with the highest need^17^. To our knowledge, there are no published evidence-based guidelines that define a standardized prioritization system for GFS, and prioritization is largely based on individual clinical judgement. A formal prioritization system could greatly improve equity and transparency in surgical access and represents an important area for future research.

Moreover, additional surgery-related variables such as glaucoma surgeons’ availability, access to operating room slots, diagnostic capacity, and staffing levels were not available in the medical records and could not be reliably reconstructed retrospectively. Therefore, these variables could not be analyzed, although they likely contributed to waiting time and inter-center variability.

On the other hand, it may be speculated that, since the multivariate model includes several patient- and procedure-related factors that significantly influence waiting times, it may partially mitigate the effect of unmeasured organizational variables. In addition, the type of anesthesia was unlikely to influence the surgical delay, as glaucoma surgery in our country is routinely performed under local anesthesia, with general anesthesia reserved only for exceptional circumstances.

Fifth, the retrospective design of the study may have introduced variability in documentation and potential information gaps, as the data were extracted from medical records. However, because we aimed to collect a large amount of preliminary information from a substantial sample (633 patients), a retrospective approach was necessary. Nonetheless, the key demographic, clinical, and surgical variables were consistently available across all patients, thereby limiting the risk of missing-data bias. However, prospective studies with standardized protocols are warranted to strengthen these preliminary findings. Sixth, the enrolment strategy based on the first fifty eligible patients per period may have introduced selection bias, particularly because we were not aware whether surgical prioritization criteria were applied and, if so, whether they varied across centers or years. Therefore, although this strategy ensured consecutive inclusion, it may not fully represent the overall surgical volume. Seventh, we did not consider the distance between the patient’s home and the hospital. Although this information was not available in our datasets, commuting distance might influence a patient’s ability to attend pre-operative appointments or accept early surgical dates. The potential impact of transport availability on surgical access deserves further investigation.

Finally, since all centers were tertiary, high-volume, public academic hospitals within the Italian healthcare system, the generalizability of these findings to private institutions, public but non-academic centers, non-tertiary services, or international settings may be limited. Thus, broader multicenter studies involving secondary-level institutions and private practices would be valuable to further extend external validity.

In closing, the strengths of this study are represented by the unicity of the topic investigated, the vast sample size, and the high translational potential of results. These results indicated that in Italy, in highly glaucoma-specialized Centers, patients with uncontrolled glaucoma waited more than forty days before undergoing GFS, with the wait for pre-surgical workup being the main time-consuming step. Moreover, despite the diffusion of faster surgical procedures, the waiting times did not change in the last quinquennium, probably because this opportunity stimulated a concomitant increase in demand. Since we found that particular geographic-, clinical-, and surgical-related conditions impacted on duration of the entire pre-surgical path, strategies taking into consideration these conditions and acting in the pre-surgical workup step, should be adopted to avoid further progression of the disease, after the surgical choice was made. Reducing surgical delay remains a significant challenge for healthcare systems worldwide.

## Methods

This multicentric retrospective study was conducted in four Italian academic Glaucoma Centers (Chieti, Genoa, Milan, and Pisa) between September 2023 and April 2024.

The study protocol was approved by the institutional review board (Department of Medicine and Ageing Science of the University ‘G. d’Annunzio’ of Chieti-Pescara, Chieti, Italy; DMSI/2023 − 101) and adhered to the tenets of the Declaration of Helsinki. Before enrollment, all patients were asked to provide a written informed consent.

Inclusion criteria for the study were: patients with primary or secondary open angle glaucoma (≥ 18 years) with uncontrolled IOP (> 22 mmHg, 9 AM), or intolerant to topical medical therapy and/or with evidence of glaucomatous damage progression, who received a formal indication for GFS and were subsequently placed on a surgical waiting list, were included for revision of their medical records. Damage progression was evaluated through trend analysis of the Humphrey Field Analyzer (II or III) Guided Progression Analysis software. Eligible procedures comprised primary or secondary filtration surgeries, performed as stand-alone procedures or in combination with phacoemulsification. Exclusion criteria were: (i) previous ocular surgery other than GFS or phacoemulsification; (ii) incomplete or missing key timing data along the pre-surgical pathway (date of indication, pre-surgical workup, or time of surgery); (iii) emergency surgeries not following the standard elective pathway; iv) Patients who received a surgical indication at one of the participating centers but subsequently underwent surgery elsewhere were excluded, as complete and comparable waiting-time data were not available.

Each Center enrolled the first fifty patients undergoing glaucoma filtration surgery and the first fifty patients undergoing re-intervention within the study periods spanning the quinquennium 2017–2022, starting from February 2017, February 2019, and February 2022. This enrolment strategy (fifty patients per center and per period) was adopted to ensure consecutive sampling. The choice of years was arbitrary but guided by methodological considerations, namely the selection of evenly spaced and representative time points while excluding the COVID-19 pandemic-affected period (2020–2021).

Clinical information was obtained from outpatient and surgical medical charts, whereas missing data was collected by inviting patients to an in person consultation.

Outcome measurements were (i) the waiting time between indication for surgery and date of surgery (main), and (ii) factors that conditioned duration of the pre-surgical path, and (iii) differences in waiting times in the last quinquennium (secondary). Waiting times were calculated in calendar days.

Patients were entered into the waiting list only when the surgical indication was formally confirmed, and the informed consent signed. Patients who wished to take additional time to reflect on the recommendation were not listed until they returned and provided written consent; therefore, the indication date coincided with the date of consent. Any decision-making interval preceding consent was not included in the waiting-time calculation.

### Waiting times between indication for surgery and date of surgery

Three time points were considered: (i) date of the indication for the first or second surgery, (ii) date of the first or second pre-surgical workup, and (iii) date of access into the operating room. These time points generated three waiting times for both first and second surgery: “*indication to surgery*”, “*indication to pre-surgical workup*”, and “*pre-surgical workup to surgery*”. Second surgery included bleb needling, bleb revision, additional GFS, or long tube implantation.

At the time of indication for surgery, we recorded gender, age, presence of monocular condition (monocular patients were defined as those with functional vision in only one eye, while the fellow eye had best corrected visual acuity (BCVA) ≤ counting fingers, hand motion, light perception, or no light perception), type of glaucoma (primary or secondary form), the scheduled first surgical procedure at the first access to the operating room (trabeculectomy, MIBS, tube), or the type of re-intervention, and whether the surgery was combined with phacoemulsification or performed stand-alone. The geographical site was also reported to differentiate whether surgery was performed in the North (Milan, Genoa), Center (Pisa) or South (Chieti) Italy.

BCVA, mean deviation (MD), visual field index (VFI), pattern standard deviation (PSD), perimetric stage of the disease (Anderson-Hodapp-Parrish criteria)^18^, IOP, number of IOP lowering eyedrops, systemic therapies, and comorbidities were also recorded. The medical chart was updated at each hospital access of the patient during follow-up. At the pre-surgical workup, clinical data was obtained from an additional ophthalmic examination, which updates information contained in the last outpatient access, along with systemic and anesthesiologist consultations, if needed. This record does not contain additional visual field examinations. At the day of surgery, the surgeon evaluated the anterior segment of the eye.

### Factors that affect the duration of the pre-surgical path

The following parameters were collected: age, gender, first surgery vs. reoperation, geographical region, glaucoma phenotype, surgical technique, combo vs. stand-alone procedure, presence/absence of monocularity or local/systemic comorbidities, IOP, perimetric stage of the disease.

### Differences in waiting times in the last quinquennium

Differences in the duration of waiting times between the three different periods were evaluated for the years 2017, 2019, and 2022.

### Statistical analysis

Shapiro-Wilk test was performed to verify normal distribution of waiting times. Descriptive statistics were reported as median and interquartile range (IQR) [first quartiles; third quartiles] data with a non-normal distribution. Categorical data were summarized as absolute frequency and percentage. Statistical differences of waiting times variables between independent groups were evaluated using Kruskal-Wallis test or Mann-Whitney U test when appropriate. Non-parametric Dunn test was applied to pairwise comparisons. Linear multiple regression model was applied to identify the potential independent predictors of the waiting time “*indication to surgery*”. Multicollinearity of the model was checked using the correlation of the independent variables and also the variance inflation factor to remove bias or confounder. Variables included in the regression model had a significant level of *p* < 0.10 both in Mann-Whitney U test or Kruskal-Wallis H test. All tests were two-tailed, with a significance level set at *p* ≤ 0.05. Statistical analyses were performed using R software environment for statistical computing and graphics (version 4.2; http://www.r-project.org/).

## Data Availability

The datasets generated during and/or analyzed during the current study are available from the corresponding author on reasonable request.
